# Development and validation of an LC-MS/MS method to quantify ceftaroline in microdialysate samples from plasma and brain: Application to a preclinical pharmacokinetic investigation

**DOI:** 10.1016/j.heliyon.2023.e16564

**Published:** 2023-05-21

**Authors:** Victória Etges Helfer, Bruna Bernar Dias, Graziela de Araújo Lock, Caroline Andrade Tomaszewski, Lucas Suchecki Barnet, Fabiano Barreto, Alexandre Prehn Zavascki, Bibiana Verlindo de Araújo, Teresa Dalla Costa

**Affiliations:** a Pharmacokinetics and PK/PD Modeling Laboratory, Pharmaceutical Sciences Graduate Program, Faculty of Pharmacy, Federal University of Rio Grande do Sul, Porto Alegre, RS, Brazil; b Federal Laboratory of Animal and Plant Health and Inspection – LFDA/RS, Porto Alegre, RS, Brazil; c Infectious Diseases Service, Hospital de Clínicas de Porto Alegre, Porto Alegre, Brazil; d Department of Internal Medicine, Federal University of Rio Grande do Sul, Porto Alegre, Brazil

**Keywords:** Ceftaroline, Microdialysis, LC-MS/MS, Unbound concentrations, Analytical method validation

## Abstract

A bioanalytical LC–MS/MS method was developed and validated to determine ceftaroline in microdialysate samples from plasma and brain. Ceftaroline was separated using a C18 column and a mobile phase consisting of water and acetonitrile, both with 5 mM of ammonium formate and acid formic 0.1%, eluted as gradient. Ceftaroline was monitored using electrospray ionization operating on positive mode (ESI+) monitoring the transition 604.89 > 209.3 *m*/*z*. The method showed linearity in the concentration range of 0.5–500 ng/mL for brain microdialysate and 0.5–2500 ng/mL for plasma microdialysate with coefficients of determination ≥0.997. The inter-and intra-day precision, the accuracy, and the stability of the drug in different conditions were in accordance with the acceptable limits determined by international guidelines. Plasma pharmacokinetics and brain distribution of the drug were carried out after intravenous administration of 20 mg/kg of ceftaroline to male Wistar rats. The estimated geometric mean (geometric coefficient of variation) area under the curve (AUC_0-∞_) was 4.68 (45.8%) mg·h/L and 1.20 (54.2%) mg·h/L for plasma and brain, respectively, resulting in a brain exposure of about 33% (AUC_free brain_/AUC_free plasma_). The results indicate that ceftaroline presents good penetration in the brain when considering free plasma and free brain concentrations.

## 1 Introduction

Ceftaroline fosamil is a cephalosporin recommended for the treatment of acute bacterial skin and skin structure infections and community-acquired bacterial pneumonia caused by Gram-positive and Gram-negative susceptible bacterial isolates [1]. In plasma, ceftaroline fosamil, which is a prodrug, is dephosphorylated by plasma phosphatase enzymes and rapidly and completely converted to the active metabolite ceftaroline [2].

Ceftaroline exhibits activity against methicillin-resistant *Staphylococcus aureus* (MRSA), which distinguishes it from other approved cephalosporins [3]. This fact has led to an increased interest in its use in the treatment of severe MRSA infections, such as those associated with the central nervous system (CNS) [3,4]. Promising results in animal models have demonstrated the potential of ceftaroline for the treatment of CNS infections [[5], [6], [7]]. Experiments using rabbit meningitis models have shown that ceftaroline penetrates up to 51% through the inflamed meninges [6]. In addition, in this studies it was shown that the levels of ceftaroline achieved in cerebrospinal fluid (CSF) were sufficient to provide bactericidal activity against Gram-positive and Gram-negative pathogens [5,7].

However, it is important to note that those results are based on total CSF concentrations. It is well known that only free drug concentrations, i.e., the fraction of the drug that is not bound to proteins, are capable of crossing the membranes and distributing from the circulatory system to the tissues [8]. Additionally, in the case of antimicrobials, since most pathogens are located in the extracellular fluid space, the free interstitial brain concentrations are the ones related to effect [8].

Microdialysis is a technique that enables one to characterize the free interstitial drug disposition in the tissue of interest. This technique is based on the passive diffusion of substances across a semipermeable membrane at the tip of a probe implanted in the tissue [9]. The probe is constantly perfused with a physiological solution, which results in a condition that never reaches equilibrium between free drug molecules from the tissue and the perfusion liquid. As a result, the measured concentration in the samples is usually low, representing only a fraction of the actual concentration in the tissue [9]. Therefore, one difficulty encountered in this technique is the quantification of the low drug concentrations in small sample volume because the combination of low perfusion flow rate (microliters/minute) and short sampling interval (few minutes) viewing to improve pharmacokinetic profile resolution, results in volumes in the few microliters range [10]. Furthermore, perfusion fluid is generally an isosmotic solution, used to mimic the interstitial fluid, with high content of electrolytes, which can interfere with drug ionization when the quantification is conducted by mass spectrometry. Hence, although the samples are usually clean and do not require processing before analysis, the development of an analytical method that meets those requirements is not straightforward.

Two methods are described in the literature for ceftaroline quantification in biological matrix [11,12]. Both methods analyzed human plasma samples, showed a lower limit of quantification of about 200 ng/mL, with injection volumes varying between 10 and 20 μL. One method used an on-line solid phase extraction system coupled to liquid chromatography tandem mass spectrometry (LC-MS/MS), monitoring the transition 605 > 208.1 *m*/*z* on positive ion mode, using gradient elution [12]. The other one analyzed the samples in an LC system with isocratic elution and ultraviolet detection at 238 nm [11]. Taking into account all the requisites that an analytical method should fulfill, these methods may not be adequate for quantifying ceftaroline in microdialysate samples.

In this scenario, this study aimed to develop and validate an LC–MS/MS method to quantify ceftaroline in Ringer microdialysate from plasma and artificial cerebrospinal fluid (ACF) microdialysate from brain samples. The method was used to evaluate ceftaroline brain penetration in healthy Wistar rats using the microdialysis technique.

## 2 Materials and methods

### 2.1 Materials and chemicals

Ceftaroline hydrochloride was provided by Pfizer (USA) and Ceftaroline Fosamil was purchased from Wyeth (Brazil). Acetonitrile and methanol with HPLC grade were obtained from Merck (Brazil). All other chemicals and reagents were of analytical grade and purchased from commercial sources. Ringer's solution was prepared in-house by mixing 147 mM NaCl, 2.3 mM CaCl_2_ and 4 mM KCl. Artificial cerebrospinal fluid was prepared by mixing 147 mM NaCl, 2.7 mM KCl, 1.2 mM CaCl_2_, 0.85 mM MgCl_2_. The solutions were filtered through a 0.47 μm filter prior to use. The microdialysis probes were purchased from CMA® Microdialysis (Kista, Sweden).

### 2.2 Chromatographic and mass spectrometric conditions

The analyzes were carried out on Agilent 1260 Infinity II LC system (Agilent, Germany), coupled with an API 5000 mass spectrometer (Sciex, Canada). Data acquisition and integration were performed using Analyst 1.6.2 software (Sciex, Canada).

Chromatographic separation was achieved on a Phenomenex Luna C18 column (50 × 2.0 mm, particle size 3 μm, Phenomenex, USA) with a pre-column Phenomenex C18 (4 × 3 mm, particle size 5 μm, Phenomenex, USA) as a security guard system. The mobile phase consisted of water (A) and acetonitrile (B) both containing 5 mM of ammonium formate and formic acid 0.1%. The flow rate was 0.5 mL/min in gradient mode. The gradient started with 5% of B increasing to 95% in 0.5 min, maintained in 95% of B until 2.3 min, and returning to initial conditions at 2.4 min and maintained until 4 min. A 2 min equilibrium time was applied in each analysis, resulting in a total run time of 6 min. The injection volume was 2 μL, the column temperature was kept at 40 °C and the autosampler temperature at 10 °C.

To obtain the best chromatographic conditions, tests were carried out using C18 and Phenyl-Hexyl columns, with particle sizes ranging from 3.5 to 5 μm, lengths from 50 to 100 mm, as well as mobile phase variation using methanol instead of acetonitrile and different concentrations of formate ammonium or ammonium acetate as modifiers. The selection of column length of 50 mm allowed a shorter total running time, increasing the sample processing capacity, without impact in retention time and peak shape reproducibility. Experiments using methanol, impaired peak shape with an impact in terms of reproducibility. Likewise, the use of ammonium formate showed an improvement in the peak shape at a concentration of 5 mM, when compared with ammonium acetate and acetic acid.

Ceftaroline was monitored using electrospray ionization in the positive mode (ESI+) and acquisition was performed in multiple reaction monitoring (MRM) mode. The parameters of the mass spectrometry were optimized by the direct infusion of 200 ng/mL of ceftaroline in water:acetonitrile (50:50, v/v) with 0.1% of formic acid. The response time (dwell time) applied was 50 ms for all transitions. Nitrogen was used as the nebulizer and collision gas. The following parameters were used: ion spray (IS) voltage of 5.5 kV; curtain gas 20 psi; nebulizer gas (GS1) 55 psi; auxiliary gas (GS2): 40 psi; source temperature: 500 °C. Table 1 shows the instrument settings optimized for product ions of ceftaroline, including the values for declustering potential (DP), entrance potential (EP), collision energy (CE) and collision cell exit potential (CXP).

**Table 1:** Monitored transitions of ceftaroline in MRM mode.

MRM Transition (*m*/*z*)	Dwell time (ms)	DP (v)	EP (V)	CE (V)	CXP (V)
604.89 > 208.3	50	131	10	37	30
604.89 > 209.3	50	131	10	37	30
604.89 > 118.2	50	131	10	65	14

DP: Declustering Potential; EP: Entrance Potential; CE: Collision Energy; CXP: Collision Cell Exit Potential.

### 2.3 Preparation of calibration standards, quality controls and sample processing

A stock solution of ceftaroline was prepared in each day of analysis by dissolving the drug in methanol on a volumetric flask to obtain a final concentration of 1 mg/mL. A series of working solutions were then prepared by dilutions of the stock solution in Ringer or ACF to obtain the final concentrations of the standard curve at 0.5, 1, 2.5, 5, 10, 25, 50, 100, 250, and 500 ng/mL for ACF and 0.5, 1, 2.5, 5, 10, 25, 50, 100, 250, 500, 1000, 2000, and 2500 ng/mL for Ringer. Quality control (QC) samples were prepared at low (1.5 ng/mL for both ACF and Ringer; LQC), medium (40 and 75 ng/mL for ACF and Ringer, respectively; MQC), and high (400 and 1500 ng/mL for ACF and Ringer, respectively; HQC) concentrations. All samples were diluted 1:1 using the ACF or Ringer's solutions and directly injected into the chromatographic system without the need of internal standard or cleanup process.

### 2.4 Method validation procedure

The bioanalytical method was validated in compliance with the US Food and Drug Administration (FDA) guidelines for biological method validation [13]. The performance parameters evaluated were selectivity, linearity, lower limit of quantification, precision (intra and inter-assay), accuracy and sample stability.

#### 2.4.1 Selectivity and carryover effects

The selectivity of the bioanalytical method was determined through the comparison of the chromatograms of the microdialysate blank samples (Ringer and ACF solutions) with the corresponding spiked samples at different concentrations of ceftaroline to determine if matrix components interfere with analyte retention time. The carryover effect was determined after successive injections of the highest concentration of the standard curve sample, followed by injections of mobile phase. After these injections, the chromatograms were observed, and the presence of peaks related to the drug was evaluated. Carryover should not be greater than 20% of the lower limit of quantification [13].

#### 2.4.2 Linearity

Linearity between the concentration and peak area was evaluated for the six analytical curves of ceftaroline in Ringer (0.5–2500 ng/mL) and ACF (0.5–500 ng/mL), produced on two consecutive days.

The residual histogram and the coefficient of determination (r^2^) of the calibration curve, as well as the relative standard deviations for the calibration curve concentrations were used to determine the homogeneity of variance between the lower and upper points of the calibration curve. Because heteroscedasticity was observed in the peak area vs. concentration curve, a weighted calibration curve was examined to evaluate the weights of 1/x and 1/x^2^. The weighting method adopted was the simplest and provided the lowest absolute sum of relative standard errors, a reasonable correlation coefficient (r^2^ > 0.98), and a relative standard deviation of no more than 20% for the lower limit of quantification (LLOQ) and no more than 15% for the other points on the curve [13]. The LLOQ was defined as the lowest concentration on the standard curve.

#### 2.4.3 Precision and accuracy

Intra- and inter-day precisions and accuracy of the method were evaluated, on three days, through the analysis of five replicates/day of QC (LQC, MQC, HQC) and the LLOQ samples in ACF and Ringer's solutions. Precision was calculated as the relative standard deviation (R.S.D.%) of intra- and inter-day QC samples responses. Accuracy was calculated as relative error (R.E.%), based on the comparison between experimental and nominal sample concentrations. Precision and accuracy should be within ±15% except for the LLOQ where it should not exceed ±20% R.S.D [13].

#### 2.4.4 Stability

Stability studies were performed in triplicate for the lowest and highest concentrations of QC samples after three freeze-thaw cycles (−80 °C ± 1 °C/room temperature); after short-term storage (samples maintained for 4 h at room temperature); after long-term storage (samples maintained for 30 days at −20 °C); and in the autosampler for 12 h at 10 °C). Samples were considered stable if the deviation from the original concentrations were within ±15% [13].

### 2.5 Microdialysis

Microdialysis was used to access free plasma and brain ceftaroline concentrations. For plasma microdialysis, a CMA® 12 concentric microdialysis probe (3 mm, PAES membrane, 20 kDa cutoff; CMA®, Sweden) was used, while for brain microdialysis a CMA® 20 microdialysis probe (4 mm, 20 kDa cutoff; CMA®, Sweden) was used. The microdialysis system consisted of a CMA® 120 system for freely moving animals (CMA®, Sweden), a PHD 2000 syringe pump (Harvard Apparatus, Holliston, USA) connected to plastic syringes (Descarpack). A FEP Tubing (1.2 μL/100 mm length; CMA®, Sweden) was used to connect the probe to the syringe and allow the probe's perfusion with ACF or Ringer solutions.

Ceftaroline probe recovery *in vitro* was carried out using dialysis and retrodialysis methods to validate the microdialysis system before the *in vivo* studies [9]. For the determination of the recovery by retrodialysis, the CMA® 12 probe was continuously perfused at 2 μL/min with a solution containing 30 ng/mL of ceftaroline and was inserted in a flask containing ACF solution at 37 °C ± 1 °C without the drug. The same procedure was performed for the CMA® 20 probe, except that the ACF solution was replaced with Ringer's solution. The system was allowed to equilibrate for 1 h, and four samples were collected with fixed collection intervals of 15 min. For the determination of *in vitro* recovery by dialysis, a similar procedure was used, but the probes were perfused with solutions without ceftaroline and placed in the flask containing the corresponding solutions with ceftaroline 30 ng/mL at 37 °C ± 1 °C. The conditions for the system, stabilization and sampling time were the same as described for the retrodialysis calibration. The relative recovery by dialysis (RR_D_) and retrodialysis (RR_RD_) were calculated using specific equations previously reported [9].

The *in vivo* calibration was conducted by retrodialysis using Ringer or ACF solutions containing ceftaroline at 100 ng/mL at a flow rate of 2 μL/min, as described in 2.6.

### 2.6 Pre-clinical pharmacokinetic study

Animal experiments were approved by Federal University of Rio Grande do Sul Ethics Committee in Animal Use (CEUA/UFRGS #35635) and were conducted in compliance with the principles of laboratory animal care from the National Council for Animal Experimentation (CONCEA/Brazil). Male Wistar rats weighing 200–250 g were obtained from the Center for Laboratory Animals Reproduction and Experimentation of Federal University of Rio Grande do Sul (CREAL/UFRGS, Porto Alegre, Brazil). The animals were kept under controlled conditions (12 h light/dark cycle; 22 °C ± 1 °C; 65% humidity) and received water/food *ad libitum*.

For the microdialysis experiments, animals (n = 6) were anesthetized with ketamine/xylazine (100 and 10 mg/kg, respectively, intraperitoneally). A CMA® 20 probe was inserted in the right jugular vein and its outlet was passed subcutaneously and exteriorized in the posterior surface of the neck. Before and after insertion, the probes were perfused for 10 min with heparinized Ringer's solution (100 IU/mL heparin) in a flow rate of 2 μL/min to prevent blood clotting in the dialysis membrane. Using a stereotaxic apparatus, a microdialysis guide cannula was surgically implanted into the primary motor cortex of the animals (A: +2.2 mm, L: +2.8 mm, V: −3.6 mm relative to bregma) [14] and fixed with two screws and dental cement (Vip Flash, Brazil). The rats recovered from the surgery in individual polypropylene boxes for 48 h.

One hour before the experiment, the animals were placed in a Bowl-Cage (CMA®, Sweden) and the guide cannulas were replaced by the CMA® 12 probe. The brain and plasma probes were continuously perfused with ACF or Ringer's solution, respectively, containing ceftaroline at 100 ng/mL at a flow rate of 2 μL/min. After a 1-h equilibration period, microdialysis samples were collected at 15-min intervals for RR_RD, in vivo_ determination. The perfusion solutions were then replaced with ACF or Ringer's solutions at a flow rate of 3 μL/min for 1 h to allow the probes to wash out. The flow rate was adjusted to 2 μL/min, and after a 30-min equilibration period, the animals were injected intravenously with a single 20 mg/kg dose of ceftaroline fosamil solution via lateral caudal vein. Microdialysate samples were collected every 15 min up to 3 h after drug administration and were frozen immediately at - 20 °C and then stored at - 80 °C until the analysis.

Non-compartmental analysis (NCA) of the plasma and tissue data was performed using the PKNCA package v.0.9.5 for R software [15], where the following parameters were obtained: elimination rate constant (λ), elimination half-life (t_½_), area under the concentration versus time curve from zero to infinity (AUC_0-∞_), clearance (CL), and volume of distribution (Vd). The ratio of AUC_0-∞, free, brain_/AUC_0-∞,free, plasma_ (tissue penetration factor – *f*_T_) was calculated as a measure of drug penetration into the brain.

## 3 Results and discussion

### 3.1 Method validation

#### 3.1.1 Selectivity and carryover effects

**Fig. 1:**
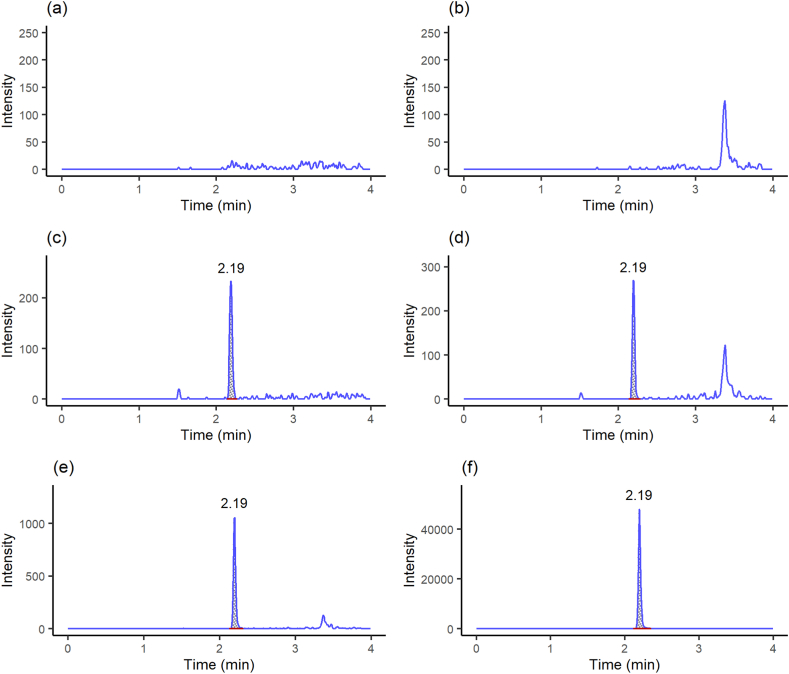
Representative chromatograms obtained from: (a) Ringer's solution, (b) ACF solution, (c) Ringer's solution spiked with ceftaroline 0.5 ng/mL, (d) ACF solution spiked with ceftaroline 0.5 ng/mL, (e) Plasma microdialysate sample obtained 2.25 h after 20 mg/kg ceftaroline i.v. administration to Wistar rats, (f) Brain microdialysate sample obtained 2.25 h after 20 mg/kg ceftaroline i.v. administration to Wistar rats.

As seen in the chromatograms present in Fig. 1(a–f), the high selectivity of the method was demonstrated by the absence of interfering peaks from endogenous or matrix compounds at the retention time of ceftaroline (2.19 min). The absence of a carryover effect was also confirmed by the absence of peaks at the same retention time of the drugs according to the previously described carryover protocol.

#### 3.1.2 Linearity

Based on the residual errors, coefficient of determination (r^2^), and best estimate of the calibrators' back-calculated concentration, a weighting scheme of 1/x was chosen for the linear regression analysis of the standard curves. The linearity was observed in the six analyzed calibration curves for both matrices, with r^2^ values above 0.997. All back-calculated values of the individual calibration standards were within 15% of the spiked value, except the LLOQ, which was within 20%.

#### 3.1.3 Precision and accuracy

Inter- and intra-day precision and accuracy results are shown in Table 2, Table 3. Precisions (R.S.D.) of the method were within 2.54–6.58% and 3.23–16.74% for intra-day analysis and 4.44–7.79% and 5.96–12.93% for inter-day analysis for ACF and Ringer solutions, respectively (Table 2).

**Table 2:** Intra- and inter-day variation of ceftaroline in artificial cerebrospinal fluid and Ringer's solution.

Ringer's Solution					Artificial Cerebrospinal Fluid				
Intra-day variation^a^					Intra-day variation^a^				
Nominal concentration (ng/mL)	Day	Mean (ng/mL)	S.D.	R.S.D. (%)	Nominal concentration (ng/mL)	Day	Mean (ng/mL)	S.D.	R.S.D. (%)
0.5	1	0.52	0.09	16.74	0.5	1	0.46	0.03	6.05
	2	0.48	0.05	9.65		2	0.49	0.03	5.51
	3	0.50	0.06	11.39		3	0.52	0.03	6.33
1.5	1	1.45	0.14	9.53	1.5	1	1.48	0.07	4.91
	2	1.49	0.07	4.91		2	1.58	0.05	3.07
	3	1.46	0.07	4.56		3	1.47	0.06	3.90
75	1	78.66	3.74	4.76	40	1	39.01	2.28	5.86
	2	79.81	4.24	5.32		2	40.48	2.50	6.18
	3	71.02	7.70	10.84		3	39.28	2.58	6.58
1500	1	1518.63	49.08	3.23	400	1	407.00	20.20	4.96
	2	1506.01	74.17	4.92		2	406.21	10.30	2.54
	3	1642.26	88.98	5.42		3	394.83	22.15	5.61
**Inter-day variation**^**a**^					**Inter-day variation**^**a**^				
0.5		0.50	0.06	12.63	0.5		0.49	0.04	7.79
1.5		1.47	0.09	6.30	1.5		1.51	0.08	5.15
75		76.49	6.51	8.51	40		39.59	2.37	5.99
1500		1555.63	92.58	5.95	400		402.68	17.90	4.44

^a^n = 5 determinations/day; S.D., standard deviation; R.S.D., relative standard deviation.

**Table 3:** Accuracy of ceftaroline analyses in artificial cerebrospinal fluid and Ringer's solution.

Nominal concentration (ng/mL)	Range (ng/mL)	Accuracy (R.E. % - range)
**Ringer's solution**		
0.5	0.42–0.60	84.0–119.5
1.5	1.35–1.69	89.9–112.5
75	64.1–85.9	85.5–114.5
1500	1430.35–1715.1	95.4–114.3
**Artificial cerebrospinal fluid**		
0.5	0.43–0.57	86.3–114.3
1.5	1.38–1.62	91.96–107.8
40	35.6–42.5	88.9–106.3
400	378.9–431.3	94.7–107.8

R.E., relative error.

The accuracy of the method for QCs ranged between 88.9 – 107.8% and 85.5–114.5% and for LLOQ between 86.3 – 114.3% and 84.0–119.5% for ACF and Ringer solutions, respectively (Table 3). These results were within acceptable criteria for bioanalytical methods [13]. Therefore, the method was considered accurate and precise for the quantification of ceftaroline in microdialysate samples from plasma and brain.

#### 3.1.4 Stability

The stability of ceftaroline in ACF and Ringer's solution was evaluated by exposing the high and low QC samples to different storage conditions simulating working scenarios. The results are summarized in Table 4. Ceftaroline showed to be stable after three freeze-thaw cycles (−80 °C ± 1 °C/room temperature); after short-term storage (samples maintained for 4 h at room temperature); after long-term storage (samples maintained for 30 days at −20 °C); and in the auto sampler for 12 h at 10 °C.

**Table 4:** Stability of ceftaroline in artificial cerebrospinal fluid and Ringer's solution.

Stability Conditions	Nominal Concentration (ng/mL)	Mean ± SD^a^	R.S.D. (%)	Accuracy (R.E. % - range)
**Ringer's solution**				
Long term^b^	1.5	1.50 ± 0.22	14.65	85.67–114.82
	1500	1406.40 ± 73.88	5.25	89.36–99.08
Short term^c^	1.5	1.52 ± 0.04	2.88	97.92–103.08
	1500	1509.98 ± 47.51	3.15	98.01–104.17
Freeze-thaw^d^	1.5	1.51 ± 0.09	6.14	94.18–106.29
	1500	1453.47 ± 46.35	3.19	93.65–99.81
12 h autosampler^e^	1.5	1.58 ± 0.13	8.38	97.56–114.72
	1500	1532.84 ± 154.36	10.07	92.42–112.93
**Artificial Cerebrospinal Fluid**				
Long term^b^	1.5	1.55 ± 0.16	10.31	92.14–113.13
	400	407.34 ± 20.73	5.09	97.23–107.45
Short term^c^	1.5	1.44 ± 0.06	4.44	91.38–99.88
	400	393.56 ± 13.76	3.50	94.70–101.51
Freeze-thaw^d^	1.5	1.59 ± 0.03	2.03	103.80–108.00
	400	400.05 ± 51.83	12.96	86.76–112.65
12 h autosampler^e^	1.5	1.47 ± 0.16	10.60	89.74–109.85
	400	410.57 ± 17.06	4.16	99.81–107.55

a Mean of 3 replicates/QC sample.

b Long term: at −20 °C for 30 days.

c Short term: at room temperature for 4 h

d Freeze-thaw: three freeze-thaw cycles.

e 12 h autosampler: at 10 °C for 12 h; S.D., standard deviation; R.S.D., relative standard deviation; R.E., relative error.

The developed and validated LC-MS/MS method proved to be rapid, sensitive, precise, and accurate, allowing the quantification of ceftaroline in ACF (0.5–500 ng/mL) and Ringer solution microdialysate samples from brain and plasma (0.5–2500 ng/mL), respectively.

### 3.2 Microdialysis and pre-clinical pharmacokinetic study

The suitability of the LC-MS/MS to quantify ceftaroline in brain and plasma microdialysate samples was investigated by applying it in a pre-clinical study of this antimicrobial brain penetration in male Wistar rats after 20 mg/kg intravenous administration.

The mean (± standard deviation) *in vitro* recovery for the CMA® 12 probe was 14.60 ± 4.84% and 16.90 ± 3.55% by retrodialysis and dialysis, respectively. Recovery for the CMA® 20 probe was 18.30 ± 1.50% and 19.40 ± 1.91% for retrodialysis and dialysis, respectively. The results demonstrate that ceftaroline does not bound to probe tubing since there was no difference between dialysis and retrodialysis recoveries, allowing the use of retrodialysis to determine the *in vivo* relative recovery of the drug. In fact, Matzneller et al. used this approach in a human microdialysis study, confirming that our findings are consistent with the literature [16]. The RR_RD,_ _*in vivo*_ showed no significant variation between animals, presenting a mean (± standard deviation) of 17.1 ± 4.5% and 16.11 ± 6.03% for CMA® 12 and CMA® 20, respectively.

**Fig. 2:**
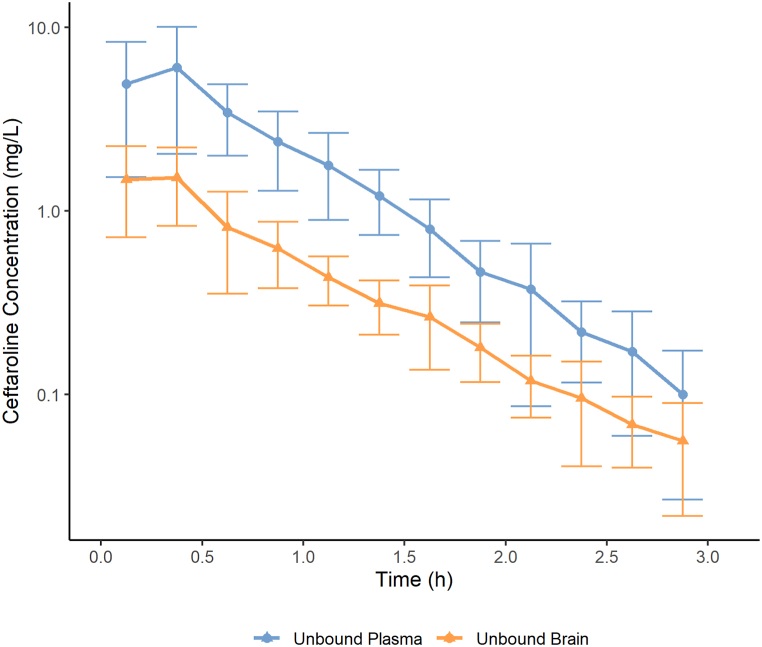
Free plasma (continuous line with closed circles) and free brain (dashed line with open circles) ceftaroline concentration after intravenous administration of 20 mg/kg to male Wistar rats (n = 6) (Mean ± SD).

Free ceftaroline plasma and brain concentration-time profiles after intravenous *bolus* dosing of 20 mg/kg to Wistar rats are presented in Fig. 2. The pharmacokinetic parameters obtained by NCA are summarized in Table 5.

**Table 5:** Ceftaroline pharmacokinetic parameters estimated by NCA after intravenous bolus administration of 20 mg/kg to male Wistar rats.

Parameter	Plasma	Brain
λ (h^−1^)^a^	1.68 (35.4%)	1.26 (23.7%)
t_½_ (h)^b^	0.431 (0.131)	0.562 (0.130)
AUC_0–3h_ (mg·h/L)^a^	4.61 (45.9%)	1.15 (54.2%)
AUC_0-∞_ (mg·h/L)^a^	4.68 (45.8%)	1.20 (54.2%)
MRT (h)^a^	0.771 (11.5%)	0.924 (27.7%)
CL (L/h/kg)^a^	4.34 (45.9%)	–
Vd_ss_ (L/kg)^a^	3.31 (53.3%)	–
*f*_T_^b^	*--*	0.33 ± 0.21

a Geometric Mean and Geometric Coefficient of Variation.

b Arithmetic Mean and Standard Deviation. $f_{T}={AUC}_{0-\infty ,free,brain}/{AUC}_{0-\infty ,free,plasma};\;n=6\;animals$

To our knowledge, this is the first work to present a pharmacokinetic analysis of ceftaroline in male Wistar rats. Ge et al. reported the plasma pharmacokinetic parameters obtained after intravenous dosing of 20 mg/kg of ceftaroline to Sprague-Dawley rats [17]. The half-life reported (0.426 h) was comparable to the one determined in this study (0.431 h). However, the reported AUC_0-∞_ was higher than the one in this study (16.3 mg h/L versus 4.68 mg h/L). Several reasons could explain this difference. First, we measured free plasma concentration, while Ge et al. determined the total plasma concentration. Second, in our study, the parameters were derived from six animals with a complete concentration-time profile, whereas, in the former analysis, the parameters were derived from a pool of three rats per collection time. Finally, there could be interspecies differences that lead to different plasma exposures [18].

The tissue penetration factor (*f*_T_, AUC_0-∞,free, brain_/AUC_0-∞,free, plasma_) revealed a mean brain exposure of 0.33 ± 0.21, meaning that approximately 33% of the free drug in plasma is able to cross the blood-brain barrier and reach the primary motor cortex of the animals. Studies that investigated the brain penetration of ceftaroline conducted in rabbits have shown a penetration of about 3% in uninflamed meninges, and up to 51% in inflamed meninges [5,6]. However, considering that these studies measured cerebrospinal fluid and total serum concentrations, a direct comparison with our study is not possible. Moreover, it is important to state that the concentrations within the brain's extracellular fluid and cerebrospinal fluid (CSF) may differ.

The correct characterization of the pharmacokinetic parameters relies on the adequate sampling and length interval of the concentration-time profile analysis. Knowing that ceftaroline is rapidly eliminated, which quickly results in low concentrations in the samples, a bioanalytical method with a low limit of quantification is crucial. The developed method showed an adequate concentration range of quantification, small injection volume, and a total analysis time suitable for a microdialysis study for ceftaroline in pre-clinical setting. Compared to the methods available in the literature, those characteristics excel, especially when considering the LLOQ and injection volume previously reported [11,12]. Although those studies were applied in the quantification of plasma samples, the LLOQ reported were 200 and 250 ng/mL with injection volumes of 10 and 20 μL, respectively [11,12]. In our study, the LLOQ was 400–500 times lower (0.5 ng/mL), with a 5 to 10 times lower injection volume (2 μL), allowing the quantification of low volume (30 μL) samples collected by microdialysis.

## 4 Conclusion

A selective, sensitive, rapid, precise, and accurate method by LC-MS/MS was validated for the quantification of ceftaroline in microdialysate samples in Ringer and ACF matrices. The method was successfully applied for the quantification of the free drug concentrations in plasma and brain of Wistar rats, with a high sensitivity (LLOQ 0.5 ng/mL) and a small injection volume (2 μL), fundamental for this type of samples. The results indicate that ceftaroline presents good penetration in brain interstitial fluid (∼33% on average) indicating that this drug should be further investigated to treat brain infections.

## Author contribution statement

Victória Etges Helfer: Conceived and designed the experiments; Performed the experiments; Analyzed and interpreted the data; Wrote the paper.

Bruna Bernar Dias: Graziela de Araújo Lock: Performed the experiments; Wrote the paper.

Caroline Andrade Tomaszewski: Lucas Suchecki Barnet: Performed the experiments.

Fabiano Barreto: Performed the experiments; Analyzed and interpreted the data; Contributed reagents, materials, analysis tools or data; Wrote the paper.

Alexandre Prehn Zavascki: Contributed reagents, materials, analysis tools or data; Wrote the paper.

Bibiana Verlindo de Araújo: Analyzed and interpreted the data; Wrote the paper.

Teresa Dalla Costa: Conceived and designed the experiments; Analyzed and interpreted the data; Wrote the paper.

## Additional information

No additional information is available for this paper.

## Data availability

The data presented in this study are available upon request to the corresponding author.

## Declaration of competing interest

The authors declare the following financial interests/personal relationships which may be considered as potential competing interests:

Alexandre Prehn Zavascki reports financial support was provided by Pfizer Inc.
